# pDNA-tachyplesin treatment stimulates the immune system and increases the probability of apoptosis in MC4-L2 tumor cells

**DOI:** 10.1007/s00726-024-03393-7

**Published:** 2024-05-01

**Authors:** Fatemeh Mahmoudi-Filabadi, Abbas Doosti

**Affiliations:** 1 Department of Biology, Faculty of Basic Sciences, Shahrekord Branch, Islamic Azad University, Shahrekord, Iran; 2 Biotechnology Research Center, Shahrekord Branch, Islamic Azad University, Shahrekord, Iran

**Keywords:** Breast cancer, Tachyplesin, Signaling pathways, Immune response

## Abstract

Breast cancer is the most common cancer among women worldwide, and marine creatures are the most abundant reservoir of anticancer medicines. Tachyplesin peptides have shown antibacterial capabilities, but their potential to inhibit cancer growth and trigger cancer cell death has not been investigated. A synthetic tachyplesin nucleotide sequence was generated and inserted into the pcDNA3.1( +) Mammalian Expression Vector. PCR analysis and enzyme digesting procedures were used to evaluate the vectors' accuracy. The transfection efficiency of MCF-7 and MCF10-A cells was 57% and 65%, respectively. The proliferation of MCF-7 cancer cells was markedly suppressed. Administration of plasmid DNA (pDNA) combined with tachyplesin to mice with tumors did not cause any discernible morbidity or mortality throughout treatment. The final body weight curves revealed a significant reduction in weight among mice treated with pDNA/tachyplesin and tachyplesin at a dose of 100 µg/ml (18.4 ± 0.24 gr, *P* < 0.05; 11.4 ± 0.24 gr *P* < 0.01) compared to the control group treated with PBS (22 ± 0.31 gr). Animals treated with pDNA/tachyplesin and tachyplesin exhibited a higher percentage of CD4 + Foxp3 + Tregs, CD8 + Foxp3 + Tregs, and CD4 + and CD8 + T cell populations expressing CTLA-4 in their lymph nodes and spleen compared to the PBS group. The groups that received pDNA/tachyplesin exhibited a substantial upregulation in the expression levels of caspase-3, caspase-8, BAX, PI3K, STAT3, and JAK genes. The results offer new possibilities for treating cancer by targeting malignancies using pDNA/tachyplesin and activating the mTOR and NFκB signaling pathways.

**Supplementary Information:**

The online version contains supplementary material available at 10.1007/s00726-024-03393-7.

## Introduction

Breast cancer is one of the most common malignancies in women. The International Agency for Research on Cancer (I.A.R.C.) reported that breast cancer was responsible for about 0.62 million fatalities in 2018 (Harwansh and Deshmukh 2020). Based on current projections, the number of occurrences is expected to rise to 3.05 million, and the death toll is estimated to reach over 7 million by 2040 (Harwansh and Deshmukh 2020). The typical treatment methods for cancer include chemotherapy, radiation therapy, and surgical intervention (Burstein et al. 2021). Traditional cancer treatment is associated with several adverse effects and often affects multiple organs. M.D.R., or multidrug resistance, may occur due to the excessive production of membrane transporters. These transporters can remove anticancer medications from within the cells, reducing drug levels and effectiveness (Sabit et al. 2022).

Moreover, radiation treatment and surgery enhance the likelihood of cancer infiltration (Harwansh and Deshmukh 2020; Burstein et al. 2021; Sabit et al. 2022). Hence, there is a need to create novel and effective medications to combat cancer (Asadipour et al. 2023; Yadollahi and Ghajari 2022). Ongoing efforts have been made to obtain potent chemicals derived from natural sources (Balandrin et al. 1985). About 71% of Earth's atmosphere is water, making it a vast source of unique bioactive compounds with unusual and distinctive chemical properties (Piri-Gharaghie 2021).

The marine ecosystem has a rich collection of anticancer compounds. In recent years, several studies have demonstrated that marine products have the potential to function as antitumor medicines and contribute to the prevention and treatment of tumors. This highlights their potential in developing new and effective medications (Yadollahi and Ghajari 2022; Balandrin et al. 1985; Piri-Gharaghie 2021). Over half of the drugs the FDA authorized in the 1980s and 1990s originated from aquatic life. Sea-derived medications are a substantial and essential source of anticancer therapies (Ahmed et al. 2021). Multiple pharmaceuticals originating from marine sources have received clearance as anticancer agents, starting with the first permission for cytarabine in 1969. Identifying Didemnin B, extracted from Trididemnum solidum in 1981 and dolastatin 10 from Dolabella auricularia in 1987, marks a significant milestone in developing cancer-fighting peptides derived from marine sources (Wu et al. 2021). Peptides derived from organic aquatic substances, such as tiny ocean peptides, have shown effectiveness as therapeutic candidates by selectively influencing several biochemical pathways (Sable et al. 2017). Marine peptides have garnered significant interest in the quest for anticancer medications due to a multitude of causes. Peptides provide many advantages compared to proteins or antibodies, including their compact size, ease of production, capacity to be easily manipulated, capability to traverse cell membranes, minimal drug–drug interactions, and versatile chemical and biological properties. Another benefit is the reduced incidence of adverse side effects from the absence of buildup in the liver or kidneys (Saeed et al. 2021; Lath et al. 2023).

Tachyplesin, a peptide of 17 amino acids, is derived from the horseshoe crab (Wang et al. 2022). The structure of this entity is amphipathic, which means it contains both hydrophobic and hydrophilic regions. This structure is achieved by the presence of two antiparallel h-sheets that are firmly kept anchored by two disulfide bonds. This structure's presence appears crucial for its antibacterial efficacy (Xie et al. 2015). In previous studies, scientists discovered that combining a synthetic form of tachyplesin with the integrin homing domain RGD effectively inhibited the proliferation of cancer cells, both in laboratory settings and in living organisms (Lu et al. 2022). RGD-tachyplesin suppressed the growth of both cultivated tumor and endothelial cells that were cultured and decreased the development of colonies in TSU cancer cells. Crucially, in living organisms, it can hinder the development of malignancies by triggering programmed cell death in both tumor and endothelial cells. Activating various caspases in both the mitochondrial and Fas-dependent mechanisms support this. Recently, researchers discovered that tachyplesin, lacking the RGD domain, may effectively hinder tumor development even in the presence of standard serum, even against cells that overexpress the multiple-drug resistance gene (Al-Benna et al. 2011). Researchers have shown that tachyplesin promotes cell differentiation and triggers apoptosis and subsequent necrosis in persistent myelogenous leukemia K 562 cells. Tachyplesin impedes the multiplication of glioma stem cells by causing damage to the plasma membrane (Kwun and Lee 2021). In addition, tachyplesin may trigger the standard complement cascade by attaching hyaluronic acid to the cell surface and C1q in the serum. This process leads to the disruption of the membrane of the cell of TSU prostate cancer cells, ultimately resulting in their demise (Hong et al. 2009). Furthermore, tachyplesin can trigger programmed cell death in glioblastoma cells when used as a nanocarrier for anti-miR210. These data indicate that tachyplesin might be used as an antitumor medication in future treatment. Nevertheless, its mechanism remains obscure (Jana et al. 2019).

We hypothesize that the potent antitumor effects of tachyplesin may be enhanced by cloning tachyplesin in a plasmid and subsequent injection into a mouse tumor model. Nevertheless, there is little understanding of the precise action mechanism of tachyplesin, and it remains uncertain how the recombinant vector of tachyplesin interacts with the membranes of the plasma of eukaryotic cells. This work aimed to clarify how the recombinant vector of tachyplesin could suppress the proliferation of tumor and endothelial cells.

## Materials and methods

### Ethics statement

The research obtained approval from the Ethics Committee of the Islamic Azad University of Shahrekord Branch in Iran (IR.IAU.SHK.REC.1402). The surgical procedures and euthanasia were carried out using intraperitoneal injections of ketamine (100 mg/kg IP) and xylazine (10 mg/kg IP). Every possible effort was undertaken to decrease the quantity of animals utilized and alleviate animal distress.

## Drugs and reagents

A solution containing 10 mg of artificially synthesized tachyplesin (CAS Number: 118231-04-2; Bachem Products, Bachem Americas, Inc.) was prepared using 10 ml of autoclaved triple distilled water. The resulting stock solution had a 1 mg/ml concentration and was stored at −20 °C. The peptide of tachyplesin, which has the sequence H-Lys-Trp-Cys-Phe-Arg-Val-Cys-Tyr-Arg-Gly-Ile-Cys-Tyr-Arg-Arg-Cys-Arg-NH_2_, was synthesized by Bachem Americas Peptides Company (Bachem Americas, USA) with a purity of 98.39%. Before use, the stock mixture was diluted to the intended final concentration using RPMI-1640 complete medium. The MTT powder from Sigma, located in MO, USA, was diluted in PBS at 5 mg/ml concentration to create a stock solution. Trypan Blue was acquired from Sigma Co. The trypsin-0.25% EDTA solution, fetal bovine serum (FBS), and antibiotic–antimycotic solution were procured from Invitrogen (Waltham, MA, USA).

## In vitro experimental

### Recombinant plasmids construction and transformation

The nucleic acid sequence was synthesized by GENEray, a commercial manufacturer in China, and then put into the pcDNA3.1 ( +) (pDNA**)** Mammalian Expression Plasmid by OriGene Wuxi Biotechnology Co., Ltd., also based in China. In this study, the gene sequence of *Tachypleus tridentatus* was selected with GenBank: M57242.1, which encodes the peptide sequence MKKLVIALCLMMVLAVMVEEAEAKWCFRVCYRGICYRRRCRGKRNEVRQIRDRGYDVRAIPEETFFTRQDEDEDDDEE. The oligodeoxynucleotide sequences were used according to Supplementary Materials 1. Concisely, the Kozak sequence was situated downstream of the CMV promoter. The recombinant tachyplesin oligodeoxynucleotides (GenBank: M57242.1) were placed between the restriction enzymes *Bam*HI (5'-GGATCC-3') and *Eco*RV (5'-GATATC-3'). The vectors were introduced into *Escherichia coli* TOP10F cells by the Cacl_2_ technique and then isolated using the Endo-free Plasmid DNA Isolation small kit manufactured by Favorgen, a company based in Taiwan. PCR, DNA sequencing, and an enzyme endonuclease digestion test assessed the efficiency of cloning. The selection of plasmids was based on the specified antibiotic concentrations: To inhibit the growth of *E. coli*, a concentration of 100 µg/ml of ampicillin should be used. The recombinant plasmids were stored at a temperature of −20 °C.

## Confirmation of the cloning of tachyplesin into the pDNA mammalian expression vector

A PCR technique used particular primers (Table 1) to trace recombinant tachyplesin. A PCR reaction with a volume of 20 µl consists of the following components: The components used in the experiment are 100 nanograms of plasmid, 1 unit of Pfu DNA polymerase enzyme, 2 µl of 10 × PCR buffer from Yekta Tajhiz Azma in Iran, 200 micromolar dNTPs from Yekta Tajhiz Azma in Iran, 10 pmol of each primer from Cinnagen in Iran, and 100 nanograms of MgCl2. The PCR temperature protocol consists of an initial annealing phase at 95 °C for 5 min, followed by 30 cycles of 45 s at 94 °C, 45 s at 55 °C, and 45 s at 72 °C. The final expansion was completed after 5 min at a temperature of 72 °C. Following electrophoresis of the PCR result on a 1% agarose gel containing ethidium bromide, the bands were examined and documented utilizing UVI Tech (England) gel imaging technology. OriGene Wuxi Biotechnology Co., Ltd. (OriGene, China) utilized BamHI and EcoRV restriction enzymes to cleave and analyze the recombinant vector.

**Table 1:** List of specific primers used in this research

Gene	Primer	Sequence (5’––––––––––– > 3’)	TM(°C)	Product Size (bp)	Accession
Tachyplesin	Tach-F Tach-R	ATGAAGTGGTGCTTCAGAGTG CTGCATCTTCTGTAGCAGATTC	57	53	Synthetic gene
*IL-6*	IL-6-F IL-6-R	CTTGGGACTGATGCTGGTGAC TCTTTTCTCATTTCCACGATTTC	65	162	NM_001314054
*PI3K*	PI3K-F PI3K-R	GGGTTGACCCCTCACCTGAC CAGGGACCACAGGGACACAG	64	153	NM_001350234.2
*AKT1*	AKT1-F AKT1-R	CGAGGTGCTGGAGGACAATG TGAGCAGCCCTGAAAGCAAG	64	200	NM_005163.2
*TSC*	TSC-F TSC-R	CTCCTCCAGCCCCACTCTGT CTGCCAGGTGGCTCTTCTGA	64	166	NR_176216.1
*mTOR*	*mTOR*-F *mTOR*-R	CATGGAGGGAGAGCGTCTGA TGAGGCCTTGGTGAGAGCTG	64	200	NM_001386500.1
*JAK*	JAK-F JAK-R	GCACTCATGGCACCTCCAAG CCAAGTGGTCCCCAAAGGAG	64	154	U09607.1
*STAT3*	*STAT3-F STAT3-R*	TGCCCCATACCTGAAGACCA ATAGCCCAGGGGCTTCCAAC	64	152	AF332508.1
*BCL2*	BCL2-F BCL2-R	GTGGATGACTGAGTACCT CCAGGAGAAATCAAACAGAG	64	118	NM_009741.5
*VEGF*	VEGF-F VEGF-R	CAGGCTGCTCTAACGATGAA CAGGAATCCCAGAAACAACC	64	164	NM_001025257
*Bax*	Bax-F Bax-R	AGGTCTTTTTCCGAGTGGCAGC GCGTCCCAAAGTAGGAGAGGAG	65	234	NM_001291431
*Caspase8*	Cas8-F Cas8-R	CACTTTCTGGGCACGTGAGG GGAACTTGAGGGAGGCCAGA	64	150	NM_033355.4
*Caspase3*	Cas3-F Cas3-R	CCGGCAAACCCAAACTCTTC CCTTGGAATTTCGCCAGGAG	64	177	AB090246.2
*GAPDH*	GDH-F GDH-R	GCCAAAAGGGTCATCATCTCTGC GGTCACGAGTCCTTCCACGATAC	64	183	NM_002046

## Cell culture and transfection

The MC4-L2 cell line, a mouse mammary cancer cell line generated from BALB/c mice and characterized as HER2 negative and ER positive, and MCF-7 and MCF-10A normal cells were obtained from the Iranian Biological Resource Center (IBRC) in Tehran, Iran. The cells were cultured in a T75 flask using Dulbecco's Modification of Eagle's Medium (DMEM)/Ham's F12 medium from Gibco/Invitrogen, located in Carlsbad, CA, USA. 2 mM L-glutamine, 15 mM HEPES buffer growth medium, 10% heat-inactivated fetal bovine serum (FBS), 100 IU/mL penicillin, and 100 mg/mL streptomycin (Gibco/Invitrogen) were added to the cell media as supplements. The flask holding the cells was placed in an environment with a temperature of 37 °C and a controlled humidity level, along with 5% carbon dioxide. When the cells reached a point where they covered around 80–90% of the flask's surface, the MC4-L2 cells were removed from the flask and then rinsed twice for 5 min each with phosphate-buffered saline (PBS).

The transfections were performed by introducing 5 × 10^4^ cells onto six-well tissue culture plates with 1μg of the appropriate plasmid. The manufacturer's instructions accomplished this using the Lipofectamine 2000 reagent from Invitrogen, Carlsbad, CA. Stable cell lines were created by selecting colonies using a 500 μg/ml G418 concentration from Invitrogen over two weeks. During the selection process, the cultural media saw daily fluctuations. The assessment of the cell proliferation rate included the initial placement of 5 × 10^4^ cells (unless otherwise specified) on six-well plates. Following 4 days, the cells were observed and quantified using a light microscope, using the Trypan Blue exclusion experiment.

## MTT assay

The MTT test evaluated the impact of pDNA/tachyplesin, tachyplesin, and PBS on the proliferation of MCF-7 and MCF10-A cells. The cell density was set to 2.0 × 10^4^/ml, and 100 μL of cells was placed in 96-well plates at a concentration of 2.0 × 10^3^ cells/well. The cells were allowed to adhere for 24 h, after which they were treated with the specified medicines. Following various durations of cell culture maintenance (in days), the cells were exposed to MTT solution (500μg/ml) for 3 h, after which the liquid above the cells was extracted. Subsequently, formazan was obtained from the pelleted cells using 100 μL of DMSO for 5 min. MTT formazan was quantified based on its absorbance at a wavelength of 570 nm.

## Colony formation assay

The cells were distributed on 6 cm diameter cultivation plates at a concentration of 1.0 × 10^2^ cells per well in triplicate. Subsequently, the cells were subjected to pharmacological treatment. Following two weeks, the cells were treated with methanol for 30 min to immobilize them. Subsequently, they were subjected to two rounds of washing with PBS. Next, the cells were exposed to amino black dye for 30 min to facilitate staining. Afterward, they were washed with running water and left to dry. Ultimately, the colonies were enumerated using a light microscope. The impact of pDNA/tachyplesin, tachyplesin, and PBS on cell proliferation was determined using the following formula:

Measured clone formation rate (%) = [number of colonies counted/number of seeded cells] × 100%.

## Transcription of apoptosis-related genes by quantitative real-time PCR

This work used a quantitative real-time PCR technique with SYBR green detection to evaluate the transcription levels of the *IL-6, PI3K, AKT1, TSC, mTOR, JAK, STAT3, BCL2, VEGF, Caspase8,* and *Caspase3* genes. According to the manufacturer's instructions, the Total RNA was isolated using the RNX TM-PLUS method (sinaclon, Iran). Subsequently, the process of cDNA synthesis was carried out employing the Parsian BioProducts Manufacturers Kit (PBP, Iran). The SYBR^®^ Premix Ex TaqTM II kit from Takara, Japan, together with particular primers listed in Table 1, was used to perform quantitative real-time PCR according to the instructions provided by the manufacturer. The relative gene transcription levels were quantified by normalizing the transcription rate of each gene to the reference gene *GAPDH*. The experiments were carried out in two distinct instances.

## In vivo experimental

### Experimental animals and tumor model

This study used female BALB/c mice, aged 5 to 6 weeks, with a body weight range of 20–23 *g*. The mice were obtained from Shahrekord Azad University in Iran. The animals were housed in a controlled environment at the animal care center of Shahrekord Azad University. Before commencing the experiment, the animals were given at least one week to acclimate to their diet and habitat. MC4-L2 cells were injected subcutaneously into the mammary fat pad of BALB/c mice at a density of 3 × 106 cells in 100 μg of sterile 1 × PBS and then allowed to develop. MC4-L2 cells were administered when their vitality exceeded 98%. The progression of the tumor was observed at intervals of 2 to 3 days, starting around 10–14 days after the HER2-/ER + MC4-L2 cells were injected. The tumors were noticeable when touched in the places where they were injected.

## Pharmaceutical formulation and design of experiments

Once the tumors grew to about 100 mm^3^, the mice carrying the tumors were divided into three groups (pDNA/tachyplesin, tachyplesin, and PBS) of 10 animals each. These groups were randomly selected to receive the vehicle. The pDNA/tachyplesin complex, tachyplesin, and PBS were dissolved in a 0.5% carboxymethyl cellulose sodium salt (CMC; Wako Pure Chemical Industries, Ltd., Japan) just before being given to the mice. The non-cancerous mice were split identically. The grouping technique is shown in Table 2.

**Table 2:** The number of mice used in this experiment

Group number	Injection composition	Number of mice	Average weight of mice	Type of injection
Cancerous group	pDNA/tachyplesin (100 µg/ml) + 0.5% CMC	10	20.7 ± 0.5	IM
	Tachyplesin (100 µg/ml) + 0.5% CMC	10	21.2 ± 0.7	IM
	PBS (100 µl) + 0.5% CMC	10	20.2 ± 0.7	IM
Non-cancerous group	pDNA/tachyplesin (100 µg/ml) + 0.5% CMC	10	22.9 ± 1.02	IM
	Tachyplesin (100 µg/ml) + 0.5% CMC	10	20.1 ± 1.22	IM
	PBS (100 µl) + 0.5% CMC	10	21.4 ± 0.68	IM

*PBS* Phosphate-buffered saline

*IM* intramuscular

*CMC* carboxymethyl cellulose sodium salt

## Tumor volume, body weight, and weight measurement

All groups' tumor sizes and body weights were observed on the initial day of medication delivery. Tumor volumes were assessed and documented at three-day intervals utilizing a digital caliper. The calculations were derived utilizing the equation *V* = (*L* × 2)/2, where *L* represents the tumor's most oversized diameter and V represents the tumor's shortest diameter. In addition, the body masses of mice were measured at three-day intervals. Following a three-week treatment period, the mice were killed. The tumor tissues were then removed and the weights of the tumors were promptly measured and recorded.

## Isolation of mononuclear cell culture from spleen, and lymph nodes

A mechanically produced single-cell suspension was obtained from the spleen and lymph nodes. The cell viability was determined to be over 98%, and all the preparation steps were performed at a temperature of 4 °C.

## Analysis of antibodies and flow cytometry

The identification of cell surface markers included the use of PerCP/Cy5.5-conjugated anti-mouse CD4, Alexa Fluor-488-conjugated anti-mouse CD8, phycoerythrin (PE)-conjugated anti-mouse CD3, and allophycocyanin (APC)-conjugated anti-mouse PD-1. Following fixation and permeabilization, the cells were internally colored with PE‐conjugated anti-mouse/rat/human Foxp3, APC‐conjugated anti-mouse CTLA-4, and APC‐conjugated anti-mouse/human Helios (an indicator of regulatory T cells derived from the thymus) antibodies. The antibodies were acquired exclusively from Biolegend Inc., located in San Diego, USA. The average number of labeled cells was determined using the FACSCalibur flow cytometer (BD Bioscience, San Jose, CA). Fluorescently labeled genotype and fluorescence minus one (FMO) standard were used to identify the likely amounts of background staining and confirm the specificity of immunostaining.

## Transcription of apoptosis-related genes by quantitative real-time PCR

This study utilized a quantitative real-time PCR method with SYBR green detection to assess the transcription rates of the *IL-6, PI3K, AKT1, TSC, mTOR, JAK, STAT3, BCL2, VEGF, BAX, Caspase8,* and *Caspase3* genes. The gene transcription levels were measured by normalizing the transcription rate of each gene to the reference gene GAPDH. The trials were conducted in two separate situations.

## Statistical analysis

The statistical analyses used GraphPad Prism version 5.0 (GraphPad Software, and Inc., USA). The flow cytometry results were analyzed using FlowJo v10.0.6 software (Tree Star and Ashland, OR). The Shapiro–Wilk technique was used to evaluate the normality of all the groupings. The statistical analysis included using the Kruskal–Wallis test and Dunn's post hoc multiple comparison analysis. The data are reported as the mean value plus or minus the standard error of the mean (SEM). A significance level of *P* < 0.05 was used for all comparisons.

## Results

### Generating and identifying recombinant pDNA/melittin

**Fig. 1:**
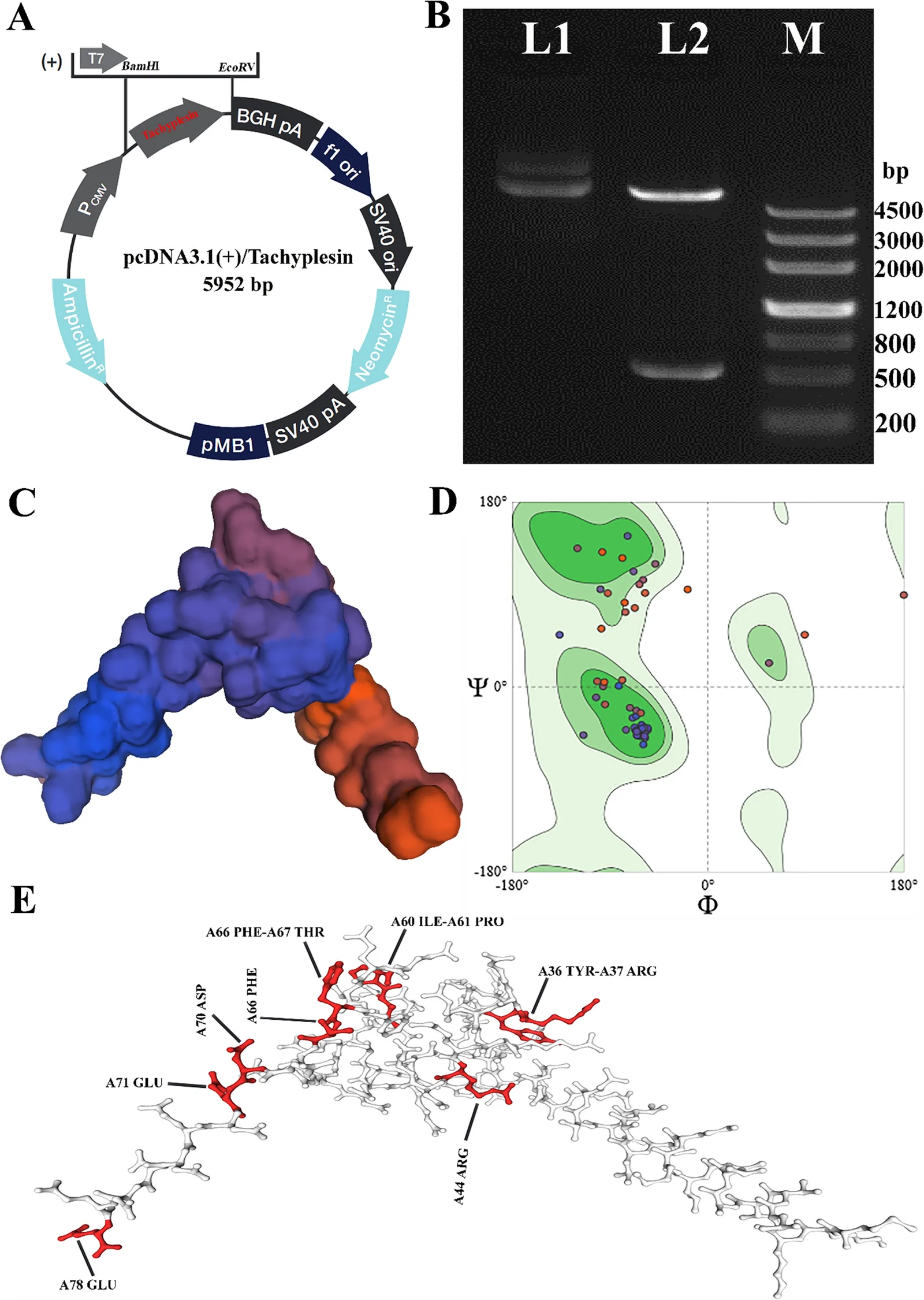
**A** Vector map of a mammalian plasmid containing pDNA/tachyplesin. **B** The successful cloning of tachyplesin into the pDNA Mammalian Expression Vector was validated using PCR. L1: The plasmid that has undergone recombination before being digested by enzymes; L2: The 524 base pair band that represents the tachyplesin gene after being digested by two enzymes; M: Marker III. **C** The three-dimensional structure of peptides. **D** The fusion peptide diagrams exhibit a Ramachandran structure with a desired outcome of 89.47%. **E** The MolProbity analysis reveals the presence of Ramachandran's bad angles

The recombinant pDNA/tachyplesin vehicle was created by including the tachyplesin oligonucleotide into the pcDNA3.1( +) expression plasmid (Fig. 1A). The genetic structure of the recombinant plasmid was used to assess the effectiveness of the cloning procedure. Supplementary Table 1 displays the complete sequencing of the recombinant vector. Furthermore, the gene sequence of the recombinant plasmid was discovered to be identical to that of tachyplesin, ascertained using DNA sequencing. The plasmid underwent digestion using *BamH*I and *EcoR*V enzymes. The validation of the formation of the recombinant plasmid was accomplished by using electrophoretic separation to separate the digestion fragments that are connected to the tachyplesin gene, specifically at a length of 524 base pairs (Fig. 1B). In addition, Fig. 1C illustrates the three-dimensional structure of the tachyplesin peptide. The Ramachandran plots of the tachyplesin peptide are shown in Fig. 1D, indicating a Ramachandran Favored score of 89.47%. Analysis of the Ramachandran chart demonstrated that all 296 bands (0/296) were within permissible criteria, indicating the absence of undesirable bands. Among the 892 angles examined, 9 angles were identified as subpar (Fig. 1E) in the specific regions: A44 ARG, A70 ASP, A78 GLU, A66 PHE, (A66 PHE-A67 THR), A71 GLU, (A36 TYR-A37 and ARG), and (A60 ILE-A61 and PRO) (Table 3).

**Table 3:** MolProbity results of tachyplesin Ramachandran plot

Analysis	Sequence	Score (%)
MolProbity score	—	1.97
Clash score	(A26 CYS-A36 TYR)	
Ramachandran Favored	—	89.47%
Ramachandran Outliers	A77 GLU, A74 ASP, A67 THR, A71 GLU	5.26%
Rotamer outliers	A69 GLN	1.43%
C-Beta deviations	A71 GLU, A78 GLU	2
Bad bonds	—	0/667
Bad angles	A44 ARG, A70 ASP, A78 GLU, A66 PHE, (A66 PHE-A67 THR), A71 GLU, (A36 TYR-A37 ARG), (A60 ILE-A61 PRO)	9/892
Cis non-proline	(A36 TYR-A37 ARG)	2/76
Twisted non-proline	(A71 GLU-A72 ASP), (A77 GLU-A78 GLU)	2/76

The evaluation of the recombinant plasmid sequencing was performed using Blast. The blast analysis (supplementary materials 2) showed that the recombinant plasmids had an exact match with the *T. tridentatus* tachyplesin mRNA gene (GenBank: M57242.1), with an E-value of 0.0, a query cover of 100%, and a 100.00% Per. Ident.

## Impact of medications on the viability of breast cancer cells

**Fig. 2:**
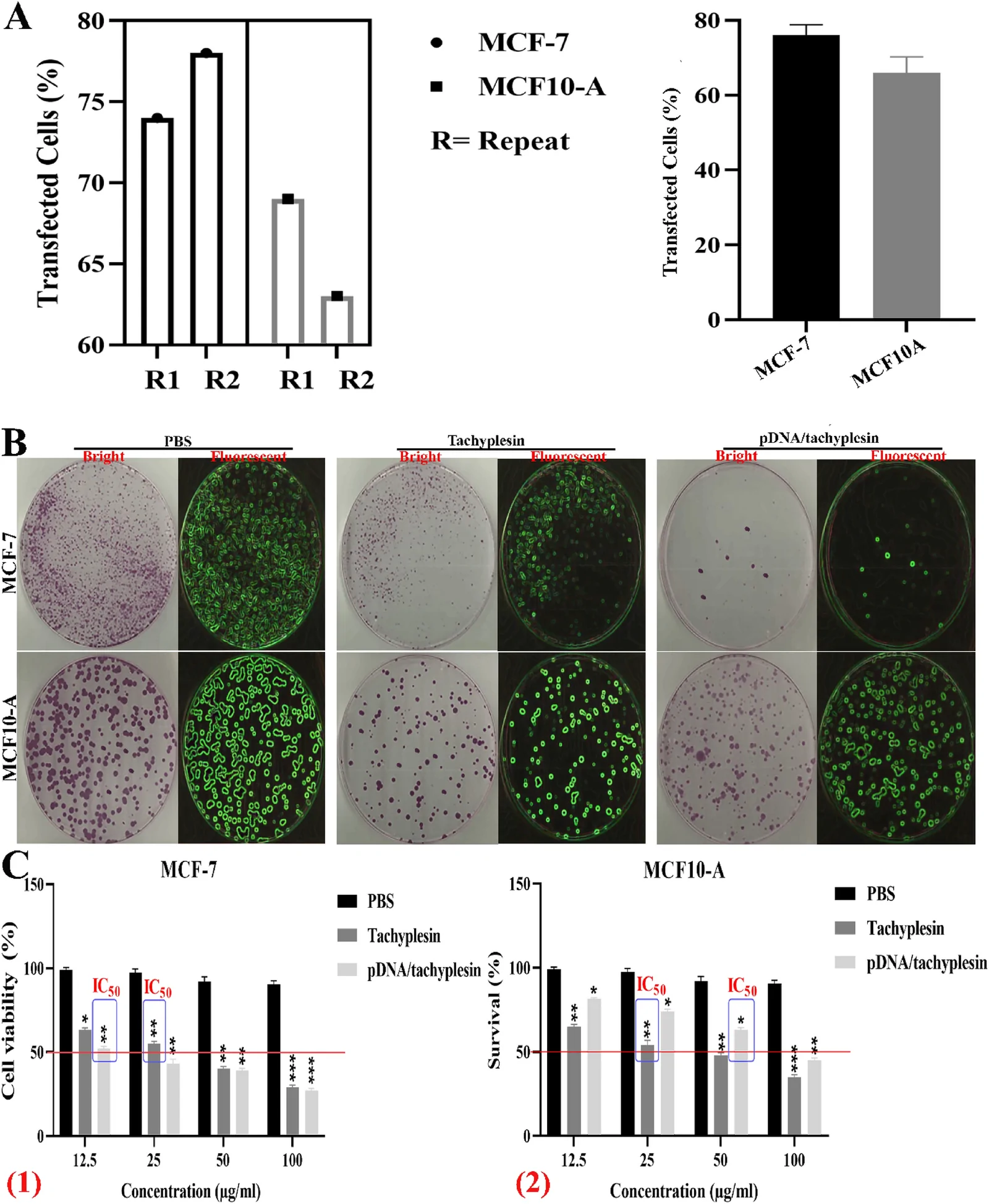
**A** Successful transfection of MCF-7 and MCF10-A cells with Lipofectamine. **B** MCF-7 and MCF10-A cell lines showed a decreased ability to form colonies following treatment with pDNA/tachyplesin, tachyplesin, and PBS. **C** Effects of pDNA/tachyplesin, tachyplesin and PBS on MCF-7 (1) and MCF10-A cells (2). The inhibitory effects of pDNA/tachyplesin, tachyplesin and PBS on MCF-7 and MCF10-A cells

MCF-7 and MCF10-A cells were subjected to transfection with pDNA/tachyplesin at a concentration of 100µg/ml (Fig. 2A). This led to a reduction in the viability of both MCF-7 and MCF10-A cells, with the extent of the decline being dependent on the dosage and duration of treatment. Experiments examining the impact of pDNA/tachyplesin, tachyplesin, and PBS on the formation of cell colonies showed that pDNA/tachyplesin significantly inhibited the ability of MCF-7 cells to multiply. Combining tachyplesin and pDNA significantly reduced the proliferation of MCF-7 cells compared to using tachyplesin alone (Fig. 2B). However, compared to the group that received tachyplesin, the group that received pDNA/tachyplesin showed a decrease in clonogenic ability in normal MCF10-A cells (Fig. 2B).

The viability of MCF-7 cells was found to be 27%, 39%, 43%, and 52% at dosages of 100 µg/ml, 50 µg/ml, 25 µg/ml, and 12.5 µg/ml per group pDNA/tachyplesin, respectively. The viability of MCF-7 cells at varying dosages of Tachyplesin is as follows: The percentage of the substance is 29% at a concentration of 100 µg/ml, 40% at a concentration of 50 µg/ml, 55% at a concentration of 25 µg/ml, and 63% at a concentration of 12.5 µg/ml. The IC50 value for the group treated with pDNA/tachyplesin was 12.5 μg/ml, whereas for the group treated with tachyplesin alone, it was 25 μg/ml (Fig. 2C). The MCF10-A cell survival rates were 45%, 63%, 74%, and 82% were exposed to dosages of 100 μg/ml, 50 μg/ml, 25 μg/ml, and 12.5 μg/ml per group pDNA/tachyplesin, respectively. The viability of MCF10-A cells, when subjected to varying concentrations of tachyplesin, is as follows: The percentage of the substance is 35% at a concentration of 100 µg per milliliter, 48% at a concentration of 50 μg/ml, 54% at a concentration of 25 μg/ml, and 65% at a concentration of 12.5 μg/ml. The IC50 value for the group containing pDNA/tachyplesin was 50 μg/ml, whereas for tachyplesin alone it was 25 μg/ml (Fig. 2C).

The results demonstrated that tachyplesin inhibited the growth of MCF-7 and MCF10-A cells via anchorage-dependent (cell proliferation) and anchorage-independent (colony formation) mechanisms. Nevertheless, the pDNA/tachyplesin treatment decreased the anchorage-dependent (cell proliferation) and anchorage-independent (colony formation) growth of MCF-7 cells. These findings indicate that tachyplesin and the mentioned pDNA might significantly enhance the vulnerability of cancerous cells. DNA plasmid increases significantly at pH 4.5. Since cancer cells have an acidic environment, the accumulation of pDNA/tachyplesin in cancer cells is higher than in normal cells. Therefore, pDNA/tachyplesin showed better IC50 on MCF-7 cells.

## pDNA/tachyplesin inhibits mTOR signaling pathway in breast cancer cells

We examined the relationship between pDNA/tachyplesin and the mTOR signaling pathway in both breast cancer cells and normal cells. The apoptotic potential of pDNA/tachyplesin was predicted using the IL-6, PI3K, AKT1, TSC, mTOR, JAK, STAT3, BCL2, VEGF, BAX, Caspase8, and Caspase3 genes. This study proposed a correlation between the stimulation of proapoptotic genes by pDNA/tachyplesin.

The upregulation of apoptotic genes in MCF-7 cells administered pDNA/tachyplesin exhibited a significant enhancement in comparison to the group administered with PBS. The transcription levels for IL-6, PI3K, AKT1, TSC, and mTOR genes in the pDNA/tachyplesin group were 0.4, 2.6, 1.9, 0.36, and 1.7, respectively. Conversely, the transcription levels in the PBS group were 0.88, 0.97, 0.74, 0.71, and 0.54, respectively. The results showed a twofold upregulation in the expression of PI3K, AKT1, and mTOR genes after treatment with pDNA/tachyplesin (**P < 0.01). Conversely, the levels of IL-6 and TSC gene expression in MCF-7 cells treated with pDNA/tachyplesin were significantly lower compared to the group treated with PBS.

In addition, after the decrease in IL-6 transcription, there was a concurrent upregulation of JAK (1.3) and STAT3 (1.8) gene expression. The upregulation of JAK and STAT3 resulted in the downregulation of BCL2 (0.32) and VEGF (0.38) transcription, as well as the upregulation of BAX (1.42), Cas8 (1.67), and Cas3 (1.38) transcription. The rate at which the JAK, STAT3, BCL2, VEGF, BAX, Cas8, and Cas3 genes were transcribed in the pDNA/tachyplesin group was 1.3, 1.8, 0.32, 0.38, 1.42, 1.67, and 1.38, respectively. The transcription rates in the PBS group were 0.88, 0.97, 0.74, 0.61, 0.83, 0.66, and 0.54, respectively, which were significantly different from the other group (*P* < 0.01).

**Fig. 3:**
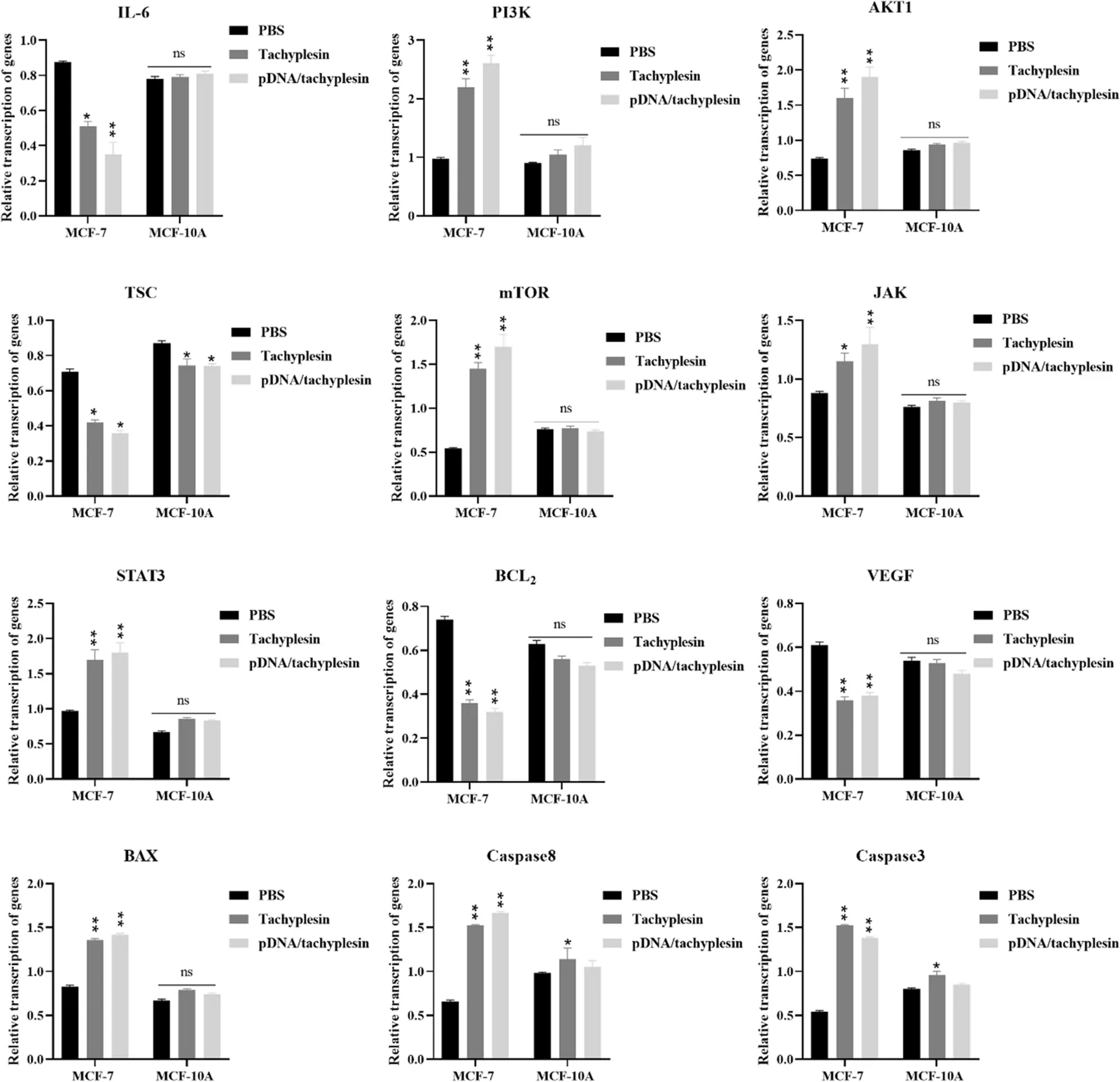
The mRNA levels of the *IL-6, PI3K, AKT1, TSC, mTOR, JAK, STAT3, BCL2, VEGF, BAX, Caspase8,* and *Caspase3* genes were evaluated in the pDNA/tachyplesin, tachyplesin and PBS control groups. The data obtained from the qRT-PCR assay were normalized versus the *GAPDH* (reference gene). *P < 0.05, **P < 0.01, *ns* non-significant

The gene transcription in the MCF-10A cells administered pDNA/tachyplesin did not show a statistically significant increase compared to the group treated with PBS. The pDNA/tachyplesin group exhibited transcription rates of 0.81, 1.2, 0.97, 0.74, 0.80, 0.73, 0.83, 0.53, 0.48, 0.74, 1.05, and 0.85 for the IL-6, PI3K, AKT1, TSC, mTOR, JAK, STAT3, BCL2, VEGF, BAX, Caspase8, and Caspase3 genes, respectively. The transcription rates in the PBS group were as follows: 0.78, 0.92, 0.86, 0.87, 0.76, 0.53, 0.67, 0.63, 0.54, 0.67, 0.98, and 0.80. The results showed a substantial increase of onefold in the expression of genes related to programmed cell death following treatment with pDNA/tachyplesin (*P < 0.05) (Fig. 3).

## In vivo experimental

### In vivo activity of pDNA/tachyplesin and tachyplesin in tumor-bearing mice

The effects of tachyplesin and the mTOR inhibitor of pDNA/tachyplesin on tumor development in mice with MC4-L2 breast cancers were initially assessed.

Upon reaching a tumor volume of around 100 mm^3^ on day 1, the mice were randomly allocated into three groups, each consisting of 10 animals. The mice were then administered PBS alone (PBS-treated animals), a high concentration (100 µg/ml) of pDNA/tachyplesin, and 100 µg/ml of tachyplesin intramuscularly three times (on days 0, 7, and 15). Administration of plasmid DNA (pDNA) containing tachyplesin and tachyplesin to mice with tumors did not result in any noticeable sickness or death throughout the therapy. Administering PBS to mice with tumors during therapy resulted in noticeable illness. No fatalities were recorded in the non-cancer groups (pDNA/tachyplesin, tachyplesin, and PBS) as shown in Table 4.

**Table 4:** Survival percentage and clinical indicators of each group

Group number	Non-cancerous group			Cancerous group		
	Body weight	Clinical score	Survival rate (%)	Body weight	Clinical score	Survival rate (%)
pDNA/tachyplesin (100 µg/ml) + 0.5% CMC	20.7 ± 0.5	−1	100%	18.4 ± 0.24	−2	100%
Tachyplesin (100 µg/ml) + 0.5% CMC	21.2 ± 0.7	−2	100%	11.4 ± 0.24	−3	100%
PBS (100 µl) + 0.5% CMC	22.2 ± 0.7	0	100%	22 ± 0.31	−4	75%

0 (normal, active, healthy), −1 (slightly sick, slightly ruffled fur, otherwise normal), −2 (ill, ruffled fur, sluggish movement, hunching), −3 (extremely sick, ruffled hair, very slow movement, stooped, eyes shut), −4 (moribund), and −5 (dead)

**Fig. 4:**
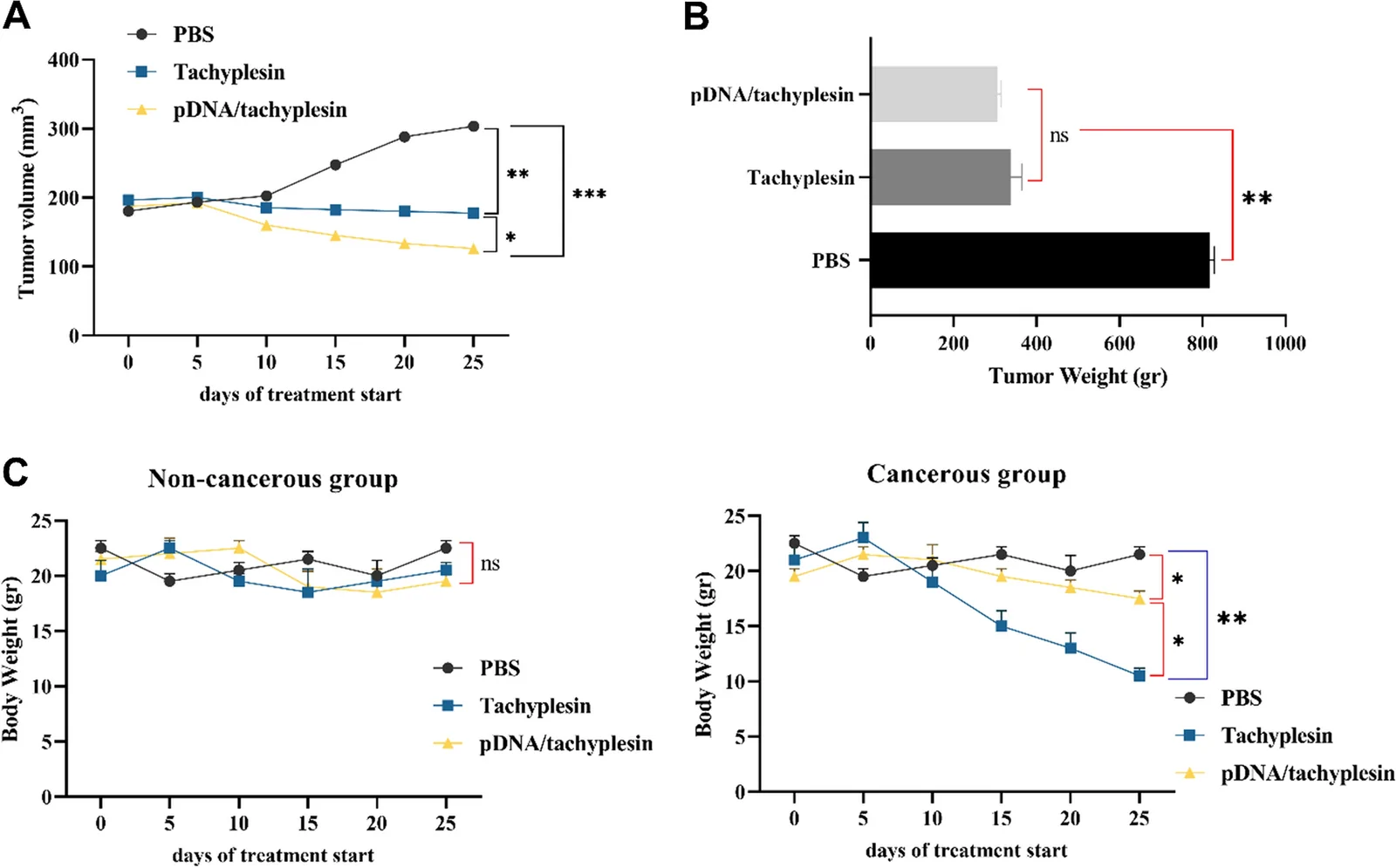
Treatment with pDNA and tachyplesin demonstrated a strong anticancer effect against mice with MC4-L2 tumors. **A** The tumor growth in mammary cancer-bearing mice is shown against time after pDNA/tachyplesin, tachyplesin, or PBS therapy. **B** After the trial, the tumor weight was significantly decreased by both the pDNA/tachyplesin and the tachyplesin therapy. **C** Body weight curves revealed that tachyplesin-treated mice significantly reduced their weight increase from the last three days of therapy, in contrast to the PBS-treated group. The information is shown as mean tumor volume ± standard deviation of the median (SEM); *P < 0.05; **P < 0.01; ***P < 0.001, using the Kruskal–Wallis test and Dunn's post hoc multiple comparisons

Our observations indicate that the administration of pDNA/tachyplesin effectively halted the development of breast tumors or shown a trend toward reduction throughout therapy. Furthermore, the efficacy of tachyplesin in inhibiting tumor development was shown to be dependent on the dosage. Our findings indicate that a concentration of 100 µg/ml of tachyplesin was particularly effective, resulting in a considerable suppression of tumor growth after 14 days of therapy (*P* < 0.001). The inhibitory impact of pDNA/tachyplesin on tumor development was statistically significant in the tachyplesin-treated groups at concentrations of 100 µg/ml (*P* < 0.001) and 100 µg/ml (*P* < 0.01) on day 21, as compared to the control animals treated with vehicle-treated PBS. Conversely, in the group that received PBS therapy, the tumor size consistently grew throughout the treatment. The tumor volume ratios after the trial were 303.5 ± 15.1 mm3 for the control animals treated with PBS, 178.4 ± 12.5 mm3 for the mice treated with 100µg/ml tachyplesin, and 125.6 ± 15.2 mm^3^ for the group treated with 100 µg/ml pDNA/tachyplesin. Figure 4A displays the tumor volume at the start and conclusion of the therapy.

To further investigate the impact of pDNA/tachyplesin on tumor development, we measured the tumor’s weight at the experiment's conclusion. The groups treated with 100 µg/ml pDNA/tachyplesin (310 ± 40 mg, *P* < 0.01) and 100 µg/ml tachyplesin (318 ± 33 mg, *P* < 0.01) showed a substantial reduction in tumor weights compared to the animals treated with PBS (824 ± 120 mg). The collected tumors were weighed after the treatment duration, as shown in Fig. 4B. Weight loss was observed in the groups treated with 100 µg/ml pDNA/tachyplesin, 100 µg/ml tachyplesin, and PBS throughout the therapy. While the mice treated with PBS consistently gained weight throughout the therapy, the groups treated with pDNA/tachyplesin began to experience weight loss around the 8th day after the mTOR inhibitor was introduced, and this weight loss was maintained throughout the treatment. The final body weight curves showed that the mice treated with pDNA/tachyplesin exhibited a significant decrease in weight at a concentration of 100 µg/ml (18.4 ± 0.24 gr, *P* < 0.05) compared to the control group treated with PBS (22 ± 0.31 gr). Conversely, the tachyplesin group at a concentration of 100 µg/ml saw a significant and substantial weight reduction of 11.4 ± 0.24 *g* (*P* < 0.01) (Fig. 4C). Therefore, apart from the suppressive impact of pDNA/tachyplesin and tachyplesin on tumor development, pDNA/tachyplesin and tachyplesin also had the potential to induce weight loss and perhaps deteriorate the animals' clinical condition. Furthermore, the degree of weight loss in the tachyplesin group with a concentration of 100µg/ml was significantly more significant compared to the group treated with pDNA/tachyplesin.

## The frequency of CD4 + Foxp3 + Treg cells and CD4 + CTLA-4 + T Cells in the spleen and lymph nodes of mice with breast tumors is increased by pDNA/tachyplesin and tachyplesin treatment

**Fig. 5:**
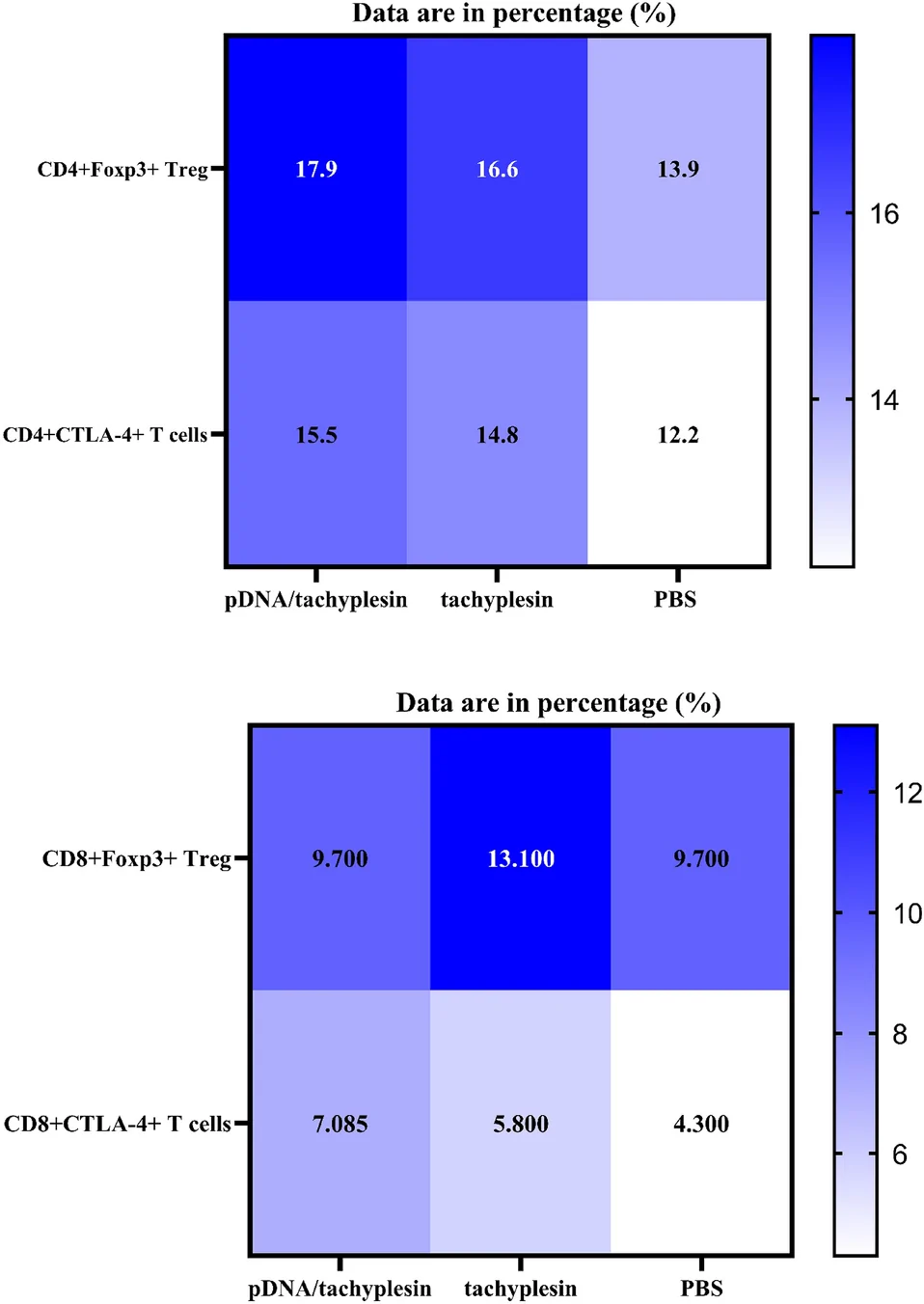
CD4 + Foxp3 + Treg cells and CD4 + CTLA-4 + T cells were analyzed by flow cytometry in the spleen of mice given treatments with pDNA/tachyplesin, tachyplesin, and PBS

Subsequently, we examined the ratio of CD4 + Foxp3 + Treg cells and CD4 + CTLA-4 + T lymphocytes in mice's spleen and lymph nodes subjected to pDNA/tachyplesin treatment and tachyplesin alone. Figure 5 demonstrates that the lymph nodes and spleen of animals treated with pDNA/tachyplesin and tachyplesin had a more significant proportion of CD4 + Foxp3 + Tregs, CD8 + Foxp3 + Tregs, and CD4 + and CD8 + T cell populations expressing CTLA-4, compared to the PBS group. This information is also summarized in Table 5. The results of our study showed that the dosage influenced the effects of pDNA/tachyplesin and tachyplesin on CD4 + and CD8 + T cells expressing Foxp3 and CTLA-4. Specifically, the administration of 100µg/ml of these substances resulted in a more significant increase in the levels of immunosuppressive substances in the lymph nodes and spleen. CD4 + and CD8 + T cells from the spleen exhibited higher levels of gMFI Foxp3 and CTLA-4 compared to the control group. Nevertheless, based on the data shown in Fig. 5, this discovery does not have enough statistical evidence to support its significance. The findings indicate a higher occurrence of Treg cells after pDNA/tachyplesin and tachyplesin treatment, which is likely to result in immune suppression and the development of tumors.

**Table 5:** The proportion of CD4 + and CD8 + expressing Foxp3 and CTLA-4 in the spleen and lymph nodes of pDNA/tachyplesin and tachyplesin-treated groups and PBS group

Group	Tissue	Subpopulation	CD4 + Foxp3 + Treg cells (Mean ± SEM)	CD4 + CTLA-4 + T cells (Mean ± SEM)	CD8 + Foxp3 + Treg cells (Mean ± SEM)	CD8 + CTLA-4 + T cells (Mean ± SEM)
Cancerous group	Spleen	pDNA/tachyplesin	17.9 ± 1.07	15.5 ± 1.03	15.1 ± 0.61	7.09 ± 0.8
		Tachyplesin	16.6 ± 0.61	14.8 ± 0.49	13.1 ± 0.48	5.8 ± 0.42
		PBS	13.9 ± 0.51	12.2 ± 0.56	9.7 ± 0.54	4.3 ± 0.41
	Lymph nodes	pDNA/tachyplesin	0.58 ± 0.16	3.21 ± 0.38	1.3 ± 0.38	1.16 ± 0.21
		Tachyplesin	0.5 ± 0.09	1.86 ± 0.43	0.46 ± 0.11	1.06 ± 0.13
		PBS	0.46 ± 0.11	1.58 ± 0.09	0.39 ± 0.09	0.99 ± 0.1
Non-cancerous group	Spleen	pDNA/tachyplesin	1.93 ± 0.04	1.97 ± 0.07	11.41 ± 0.06	1.87 ± 0.2
		Tachyplesin	1.69 ± 0.11	1.93 ± 0.04	11.23 ± 0.04	1.84 ± 0.9
		PBS	1.65 ± 0.17	1.87 ± 0.06	10.59 ± 0.04	1.69 ± 0.3
	Lymph nodes	pDNA/tachyplesin	0.83 ± 0.05	1.46 ± 0.07	0.94 ± 0.06	0.97 ± 0.3
		Tachyplesin	0.78 ± 0.07	1.32 ± 0.08	0.89 ± 0.08	0.93 ± 0.5
		PBS	0.59 ± 0.04	1.08 ± 0.04	0.67 ± 0.04	0.89 ± 0.6

*SEM* standard error of the mean

## pDNA/tachyplesin inhibits survival, autophagy, and angiogenesis in tumor tissue

**Fig. 6:**
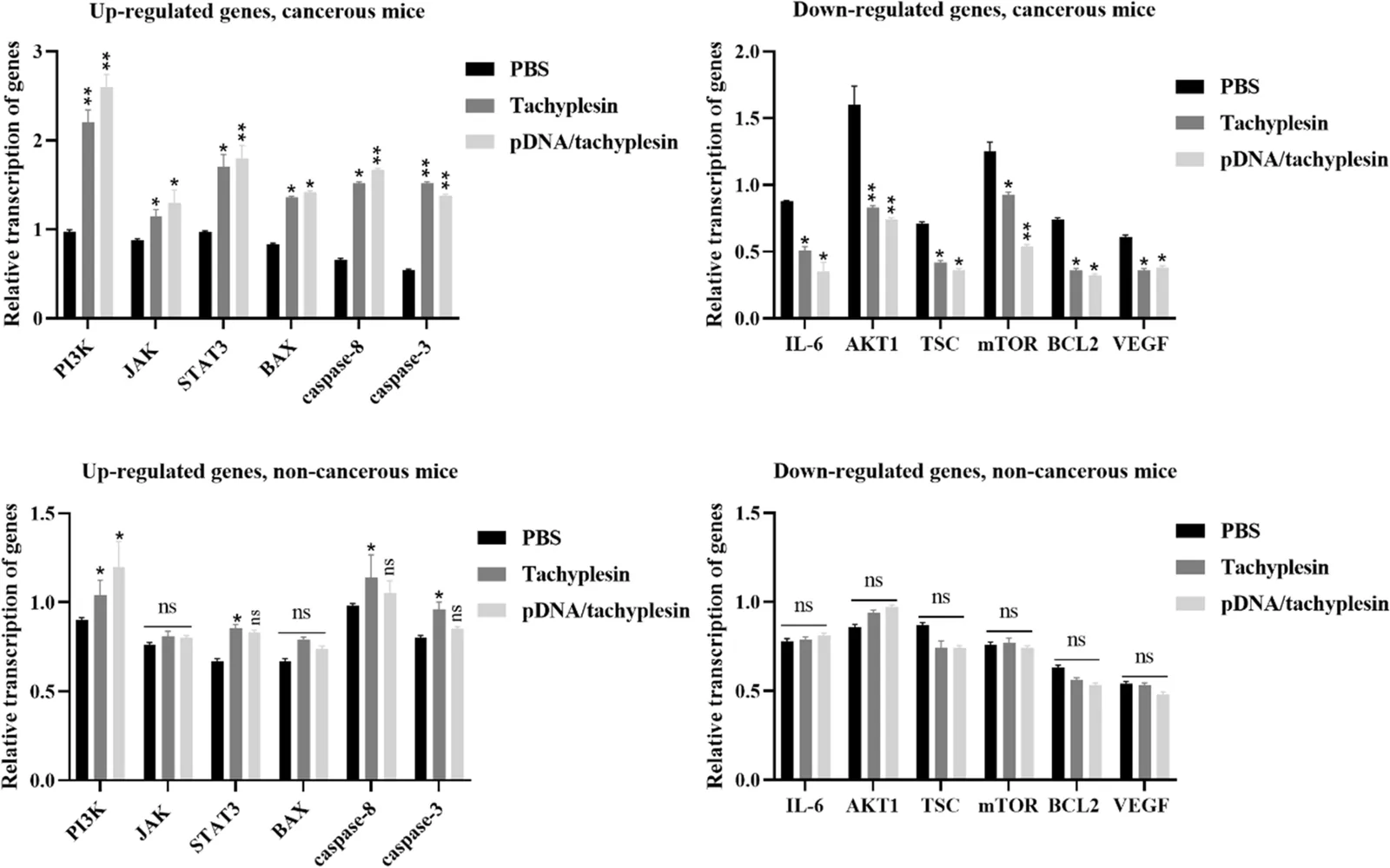
Investigating the transcriptional activation of signaling pathway genes after treatment with pDNA/tachyplesin, tachyplesin, and PBS in cancerous and non-cancerous mice

**Fig. 7:**
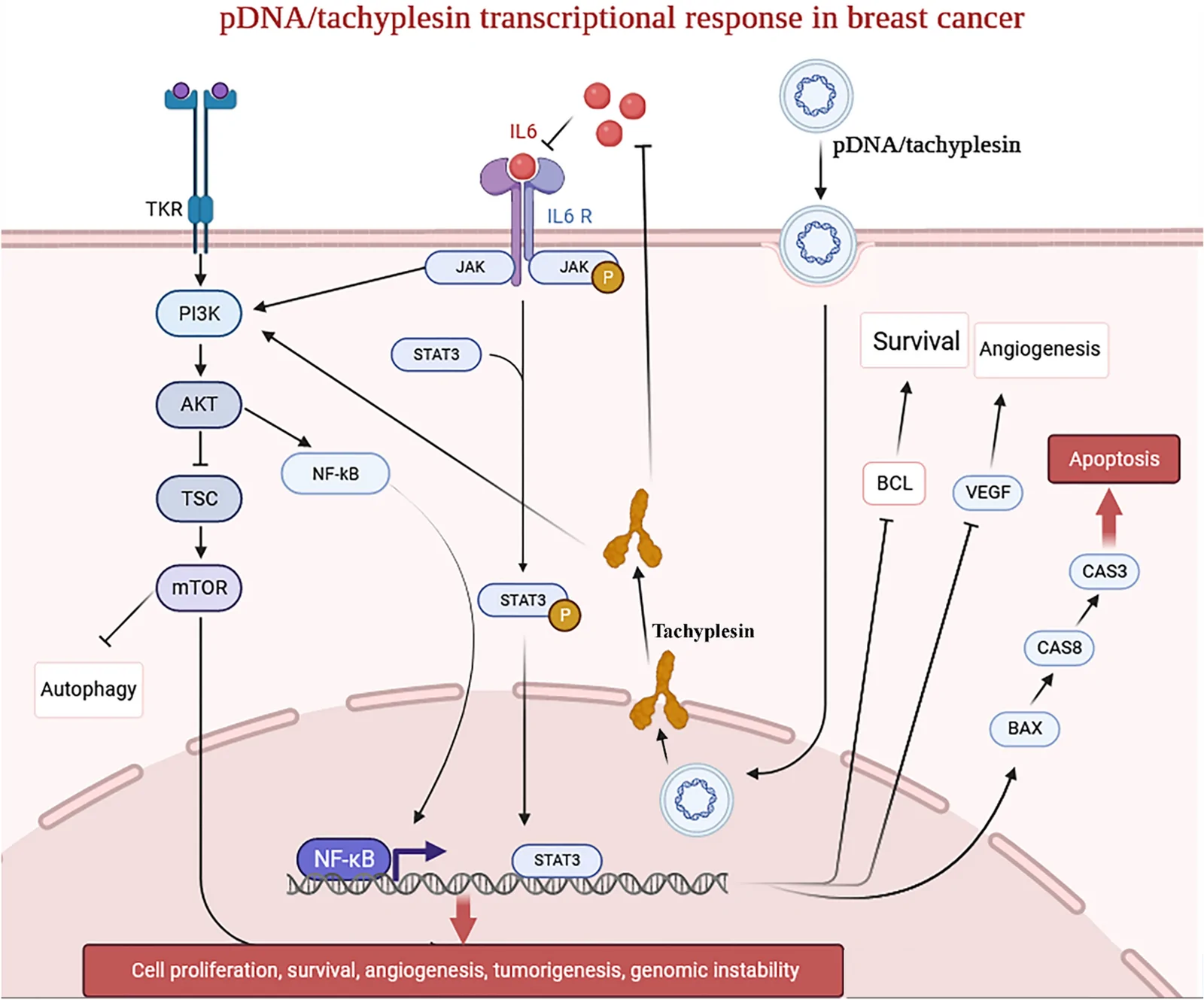
The use of pDNA-tachyplesin to treat malignancies involves the activation of the mTOR and NFκB signaling pathways

We hypothesized the existence of a new signaling pathway, including the genes IL-6, PI3K, AKT1, TSC, mTOR, JAK, STAT3, BCL2, VEGF, BAX, Caspase8, and Caspase3. The experiments on living organisms showed a direct relationship between the increase in proapoptotic transcription factors in the group treated with pDNA/tachyplesin. The groups treated with pDNA/tachyplesin showed significantly increased expression levels of caspase-3, caspase-8, BAX, PI3K, STAT3, and JAK genes (Fig. 6, *P* < 0.01). Subsequently, it was shown that the expression levels of the genes IL-6, AKT1, TSC, AKT1, mTOR, BCL2, and VEGF were downregulated in the pDNA/tachyplesin-treated group compared to the tachyplesin and PBS-treated groups. The administration of pDNA/tachyplesin did not result in a substantial reduction in the transcription levels of IL-6, AKT1, TSC, AKT1, mTOR, BCL2, and VEGF genes in the normal non-cancerous groups (Figs. 6, 7).

## Discussion

The treatment of cancer patients has involved the application of innovative chemotherapeutic substances, especially those obtained from botanical and zoological sources (Jana et al. 2019; Ahmad et al. 2021). Nevertheless, in the end, negative consequences and resistance to medicine occur. In recent years, peptides and vaccination have gained popularity as therapy approaches (Cunha et al. 2017). Tachyplesin was proposed as a potential treatment choice for treating breast cancers in this case. When exposed to the IC50 level, this compound is more beneficial than other chemicals in inducing early apoptosis in MCF-7 cells. The observed changes in the structure and appearance of cells, together with the significantly increased activity of caspase-3, caspase-8, BAX, PI3K, STAT3, and JAK genes, respectively, confirm that the use of pDNA/tachyplesin resulted in the programmed cell death of MCF-7 cells. The mortality of breast cancer BT-474 cells caused by cardanol resulted in comparable alterations in gene transcription (Su et al. 2017). However, the dose was the determining factor in whether tachyplesin induced necrosis or apoptosis.

The tumor microenvironment is crucial in determining the resistance and survival of cancer. To evaluate the process of monocyte-to-macrophage differentiation, two distinct phenotypes associated with pro- and antitumor characteristics were analyzed (Minopoli et al. 2020). Studies suggest that M2-polarized macrophages may have had a role in cancer resistance by facilitating tumor growth and survival. Therefore, AKT may be associated with M2-polarized macrophages (Wang et al. 2021). This study discovered that tachyplesin inhibited the development of neutrophils in C-PBS but not in pDNA/tachyplesin. This conclusion is rather noteworthy. The molecular mechanism behind the suppression of tachyplesin remains unclear. To determine whether Neutrophils were converted into myeloblasts, evaluating the levels of biomarkers associated with the mTOR signaling pathways is crucial (He et al. 2022). Interestingly, ß-element derived from the Chinese herb Curcuma wenyujin can induce the re-differentiation of myeloblasts into neutrophils. This process involves the upregulation of AKT1 and the BAX gene (Zhou et al. 2016).

Previous research has identified some cellular regulators that influence the spread of breast cancer cells, particularly the reorganization of the cell's internal structure necessary for cancer cell mobility. This process requires a significant quantity of ATP (Mierke 2019). Mitochondria are responsible for aerobic ATP generation. They intertwine with the other organelles to form a network of tubular structures (Luciani et al. 2021). STAT3 regulates mitochondrial respiration (Wegrzyn et al. 2009). However, even when oxygen is present, tumor cells often get a significant portion of their energy by aerobic glycolysis, which sets them apart in biological energies (Potter 2009). The functional significance of mitochondrial function in a mobile cancer cell remains uncertain due to the existence of two pathways for energy acquisition (Luciani et al. 2021; Wegrzyn et al. 2009; Potter 2009). This study is the first investigation to demonstrate the significant involvement of mitochondrial fission in the dissemination of breast cancer. By analyzing human breast cancer specimens, we observed a substantial increase in the expression of STAT3 protein in invasive breast carcinoma and tumor that had metastasized to lymph nodes. However, the elevation of STAT3 protein was only marginally higher in non-invasive cancer in situ compared to normal breast tissues. These data suggest that the first stage of developing a JAK in breast cancer phenotype may require an overexpression of STAT3. The expression of the STAT3 protein in cancerous breast specimens exhibited significant variation, even within specimens within the same category. If STAT3 overexpression is more common in a specific subtype of breast cancer, it might serve as an essential indicator for therapy planning and the development of new medications (Saha and Lukong 2022). A recent study has shown that breast cancer cell lines with variable degrees of anticancer potential have unique profiles of mitochondrial proteins (Cheng et al. 2012).

This research documented that tachyplesin can impede the growth of breast cancer cells and trigger their programmed cell death, also known as apoptosis. The findings aligned with the outcomes of other investigations conducted in different tumor models. Nevertheless, tachyplesin did not influence the migration of normal breast cells in a laboratory setting. Significantly, the findings demonstrated that combining pDNA and tachyplesin could enhance the responsiveness of breast cancer cells that were resistant to treatment. The pDNA/tachyplesin complex may cause apoptosis by generating DNA damage and cell cycle arrest via exogenous and endogenous routes, resulting in anticancer effects. The pDNA/tachyplesin combination has potent and wide-ranging anticancer properties, making it one of the most efficacious medications. Currently, it is used in clinical practice. The resistance of cancer cells to medications may occur via the inactivation of the apoptotic pathway and the activation of cellular defense mechanisms against apoptosis (Chaudhry et al. 2022). One specific method involves the overexpression of anti-apoptotic proteins like Bcl-2, which can lead to the development of drug-resistant cancer cell lines. Recent research has shown that antitumor medications may effectively counteract tumor cell resistance by promoting apoptosis (Qin et al. 2023). Our research has shown that pDNA/tachyplesin can trigger apoptosis in breast cancer cells. Moreover, we have verified that the combination of plasmid DNA (pDNA) and tachyplesin can impede the growth and movement of cancerous cells.

The MC4-L2 mammary cancer is a transplantable cancer cell line that is highly invasive and can cause tumors. It may move from the original cancer in the mammary gland to numerous distant regions (Farhanji et al. 2015). Moreover, there is a notable similarity between the progressive progression of MC4-L2 metastases in lymphatic arteries and tissues of mouse models of BALB/c and human breast tumors (Zhang et al. 2021; Valdelvira et al. 2021). Tumors were observed in untreated mice after a 14-day injection of MC4-L2 cells throughout the experiment. According to the final body weight measurements, pDNA-tachyplesin had no adverse effects. Moreover, this study demonstrated that tumors inoculated with MC4-L2 cells exhibited a size reduction that was four times lower compared to the group that received treatment with pDNA-tachyplesin. These findings indicate that using pDNA-tachyplesin treatment effectively prevented the spread of MC4-L2 cancer cells, supporting the researchers previously documented in vitro ability to limit cell invasion and migration (Ding et al. 2015).

This research has shown that activating the mTOR and NFκB pathways is crucial for controlling the inhibitory impact of tumor-initiating cells in this tumor model. This study also reveals a significant reduction in the number of mice breast tumor-initiating cells by pDNA-tachyplesin. Based on our research, the cancer cells in the control animals had fast tumor recurrence, eventually expanding to a significant size in BALB/c mice. Tumors derived from tumor cells treated with pDNA-tachyplesin had a tumor dimension that was four times smaller than tumors originating from the untreated control group. These findings indicate that tumors treated with pDNA-tachyplesin may activate the mTOR and NFκB signaling pathways, thereby accounting for the delayed development of MC4-L2 tumors in mice xenografts.

## Conclusions

Our findings provide novel possibilities for treating cancers using pDNA-tachyplesin and stimulating the mTOR and NFκB signaling pathways. Further investigation is required to thoroughly understand the complex mechanisms involved in the interactions between pDNA and tachyplesin in the mTOR and NFκB signaling pathways. The research presented indicates that pDNA-tachyplesin has the potential to be a beneficial novel therapeutic option that may be used with current chemotherapy treatments as an antiangiogenic treatment. It is recommended that studies be conducted in the areas of autophagy suppression, cancer, and genetic stability to develop a comprehensive understanding of the intricate mechanisms behind the interactions between pDNA and tachyplesin in the mTOR and NFκB signaling pathways.

## Supplementary Information

Below is the link to the electronic supplementary material.

Supplementary file1 (DOCX 15 KB)

Supplementary file2 (TXT 2 KB)

## Data Availability

The datasets analyzed during the current study are available from the corresponding author upon reasonable request.
